# C-reactive protein/albumin ratio and Glasgow prognostic score are associated with prognosis and infiltration of Foxp3+ or CD3+ lymphocytes in colorectal liver metastasis

**DOI:** 10.1186/s12885-022-09842-4

**Published:** 2022-08-01

**Authors:** Hiroki Kanno, Toru Hisaka, Jun Akiba, Kazuaki Hashimoto, Fumihiko Fujita, Yoshito Akagi

**Affiliations:** 1 Department of Surgery, Kurume University School of Medicine, 67 Asahi-machi, Kurume, Japan; 2 Department of Pathology, Kurume University School of Medicine, Kurume, Japan

**Keywords:** Colorectal liver metastasis, Inflammatory index, Tumor-infiltrating lymphocytes

## Abstract

**Background:**

Inflammatory indices and tumor-infiltrating lymphocytes (TILs) have prognostic value in many cancer types. This study aimed to assess the prognostic value of inflammatory indices and evaluate their correlation with survival and presence of TILs in patients with colorectal liver metastasis (CRLM).

**Methods:**

Medical records of 117 patients who underwent hepatectomy for CRLM were retrospectively reviewed. We calculated inflammatory indices comprising the neutrophil/lymphocyte ratio, platelet/lymphocyte ratio, C-reactive protein/albumin ratio (CAR), and Glasgow prognostic score (GPS). Furthermore, we evaluated the relationship between these ratios and the GPS and survival rates and immunohistochemical results of tumor-infiltrating CD3+, CD8+, and Foxp3+ lymphocytes.

**Results:**

The patients with low CAR values and low GPS had significantly better overall survival as per the log-rank test (*p* = 0.025 and *p* = 0.012, respectively). According to the multivariate analysis using the Cox proportional hazard model, the CAR (hazard ratio [HR], 0.57; 95% confidence interval [CI], 0.33–0.99; *p* = 0.048) and GPS (HR, 0.40; 95% CI, 0.19–0.83; *p* = 0.013) were independent prognostic factors. Additionally, Foxp3+ lymphocytes were more common in samples from the patients with a low CAR (*p* = 0.041). Moreover, the number of CD3+ TILs was significantly higher in the patients with a low GPS (*p* = 0.015).

**Conclusions:**

The CAR and GPS are simple, inexpensive, and objective markers associated with predicting survival in patients with CRLM. Moreover, they can predict the presence of Foxp3+ and CD3+ lymphocytes in the invasive margin of a tumor.

**Trial registration:**

Retrospectively registered. https://www.kurume-u.ac.jp/uploaded/attachment/14282.pdf.

**Supplementary Information:**

The online version contains supplementary material available at 10.1186/s12885-022-09842-4.

## Background

Colorectal cancer (CRC) is the third leading cause of cancer-related deaths worldwide [1]. Approximately, 33% of patients with CRC experience metastasis during clinical course, which worsens their survival. Liver is the most common site of metastasis in patients with CRC. Liver resection remains the only potential curative treatment of colorectal liver metastasis (CRLM), with a 5-year survival rate of approximately 44–49.6% [2, 3]. Nevertheless, the recurrence rate after hepatectomy for CRLM has been reported to be approximately 62% [4]. Therefore, identifying effective biomarkers is necessary for predicting CRLM recurrence risk and chances for survival.

Systemic inflammation and cancer are closely inter-related. Cancer-related inflammation can affect tumor progression, which in turn induces systemic inflammation [5, 6]. In several cancers, including CRLM, inflammatory indices such as the neutrophil/lymphocyte ratio (NLR), platelet/lymphocyte ratio (PLR), and C-reactive protein/albumin ratio (CAR) have been indicated to have good prognostic values. In addition, the Glasgow prognostic score (GPS), which assigns categorical values between 0 and 2 depending on a combination of serum levels of C-reactive protein and albumin, has also been proven to have a good prognostic value [7–11]. Thus, systemic inflammation is a key factor in tumorigenesis. However, its detailed mechanism remains unclear.

The host tumor microenvironment plays a pivotal role in cancer progression. Some studies have reported that presence of tumor-infiltrating lymphocytes (TILs) influences long-term outcomes of many cancer types [12–14]. In patients with CRC, higher numbers of CD3+ and/or CD8+ TILs are associated with better prognosis. However, a high number of Foxp3+ TILs can be indicative of either better or worse prognosis, depending on the primary tumor site and other accompanying factors [15, 16]. Although several studies have evaluated the relationship between inflammatory indices and presence of TILs [17, 18], no reports have assessed this relationship in patients with CRLM.

Therefore, the present study aimed to explore the prognostic value of inflammatory indices, including the NLR, PLR, CAR, and GPS, and evaluate their correlation with the presence of TILs in patients with CRLM.

## Methods

### Patients

The present study was approved by the Research Ethics Committee of Kurume University, Kurume, Japan (approval number: 21228) and conducted in accordance with the Declaration of Helsinki. The requirement for informed consent was waived due to the retrospective nature of the study. The medical records of 202 Japanese patients who underwent hepatectomy for CRLM at Kurume University between January 2006 and December 2019 were retrospectively reviewed. Among them, records of 85 cases were excluded: 34 cases had undergone repeated hepatectomy for CRLM and 33 cases had metastases to other sites, including the lungs, lymph nodes, and/or peritoneum. In addition, curative resection was not achieved in 5 cases. In the remaining 13 excluded cases, pathological evaluation was not performed due to missing samples. Therefore, totally, the data of pathologically confirmed 117 patients were reviewed.

### Data collection

Clinicopathological data were obtained from the patients’ medical records. Blood samples were collected during the week prior to surgery. Regarding neoadjuvant chemotherapy (NAC), we selected oxaliplatin- or irinotecan-based regimens with/without therapeutic monoclonal antibodies. Postoperative follow-up was conducted as follows: levels of tumor markers such as carcinoembryonic antigen (CEA) and/or carbohydrate antigen 19–9 were measured every 3 months after surgery. Computed tomography was performed every 3–6 months after surgery. Recurrence-free survival (RFS) was defined as the time from surgery to recurrence or death. Overall survival (OS) was defined as the time from surgery to death.

### Calculation and cutoff value of each inflammatory index

Each inflammatory index was calculated as: NLR, ratio between peripheral blood neutrophil and lymphocyte counts; PLR, ratio between peripheral blood platelet and lymphocyte counts; CAR, ratio between serum C-reactive protein (CRP) and albumin concentrations. The GPS was calculated using both CRP and albumin concentrations. The corresponding cutoff values were < 1.0 mg/dL for CRP and ≥ 3.5 g/dL for albumin. If both CRP and serum albumin concentrations were within reference values, the GPS was set to 0. If either one of them was out of reference, the GPS was set to 1. If both CRP and albumin concentrations were out of reference values, the GPS was set to 2. Median values were used as cutoff values for NLR, PLR, and CAR. Regarding each index, we categorized the patients in two groups (“low” or “high”) depending on their results with respect to the cutoff. In regard to the GPS, “low” corresponds to a GPS = 0 and “high” corresponds to a GPS = 1 or 2.

### Immunohistochemistry

Formalin-fixed paraffin-embedded tissue slides were cut in 4 μm, examined on a coated glass slide, and labeled with the following antibodies using the Bond-III autostainer (Leica Microsystems, Newcastle, UK): anti-CD3 antibody (1:300, clone LN10, Leica Microsystems, Newcastle, UK, Catalog No: CD3–565-L-CE), anti-CD8 antibody (1:200, clone 4B11, Leica Microsystems, Newcastle, UK, Catalog No: CD8-4B11-L-CE), and anti-Foxp3 antibody (1:100, clone 236A/E7, Abcam, Cambridge, MA, USA, Catalog No: ab20034). Briefly, samples for anti-CD3, -CD8, and -Foxp3 antibodies were heat-treated using epitope retrieval solution 2 (pH 9.0) at 99 °C for 15, 15, and 30 min, respectively, and then incubated with each antibody for 30 min at room temperature. This automated system used a Refine detection system (Leica Microsystems, Newcastle, UK) with a horseradish peroxidase-conjugated polymer as the secondary reagent and 3,3′ diaminobenzidine as the chromogen.

### Evaluation of TILs

**Fig. 1:**
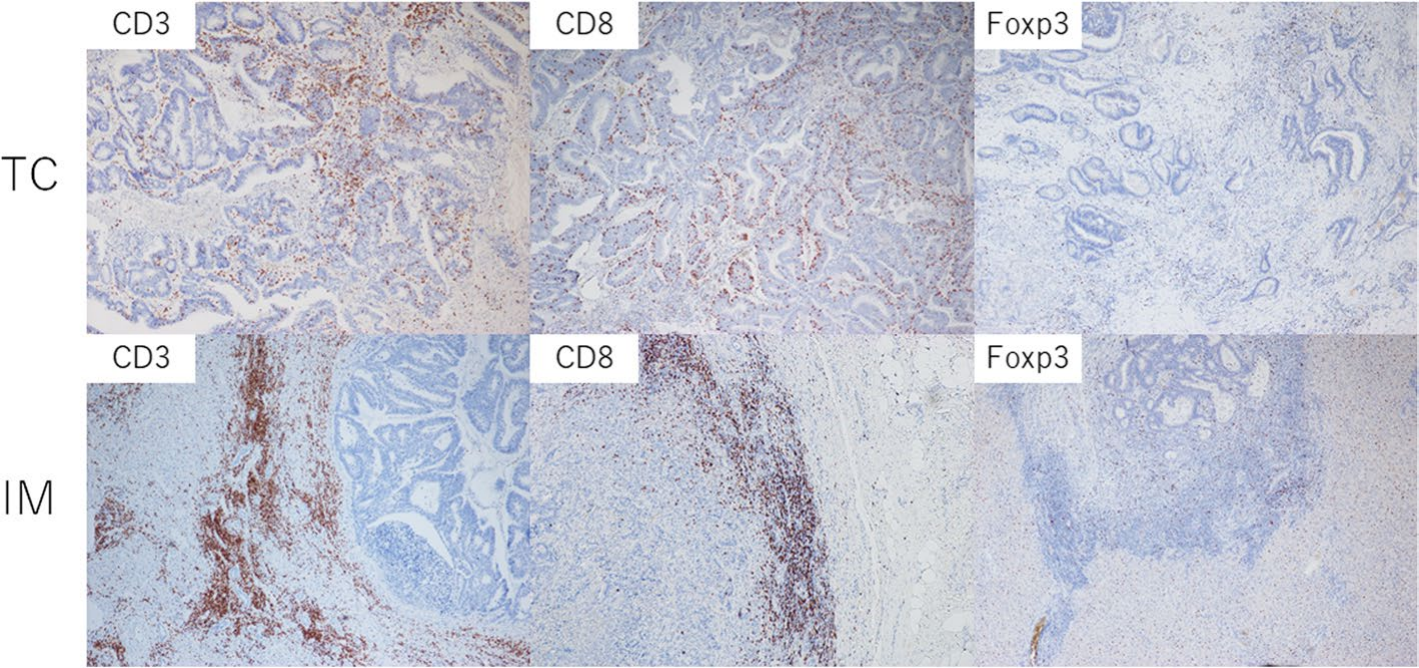
Representative immunohistochemical images of CD3+, CD8+, and Foxp3+ lymphocytes in the tumor center (TC) and invasive margin (IM)

Expression of each protein in the surrounding normal liver parenchyma was used as positive and negative internal controls in all cases. The total number of lymphocytes (expressing CD3, CD8, and Foxp3) in the tumor center (TC) and at the invasive margin (IM) was separately counted at × 400 magnification. To reduce effects of tumor heterogeneity, three well-stained spots were evaluated in each sample, and the average of the three measurements was used for analyses. Representative immunohistochemistry images are shown in Fig. 1.

### Statistical analyses

Clinicopathological characteristics in the two groups (low NLR versus high NLR, low PLR versus high PLR, low CAR versus high CAR, and GPS:0 versus GPS: 1/2) were compared using the chi-square test for categorical variables: sex, primary tumor location, NAC, onset, metastatic tumor location, and tumor number. The Mann–Whitney U test was used for continuous variables: age, body mass index (BMI), preoperative CEA level, and liver tumor diameter. There were no missing values in the present study. Recurrence-free and OS curves between the two groups were created using the Kaplan–Meier method and compared using the log-rank test. A Cox proportional hazards model was used for univariate and multivariate analyses. Moreover, model assumptions were verified by the likelihood ratio test. The variables that might affect prognosis were added into the Cox proportional hazard model, and hazard ratios (HRs) and 95% confidence intervals (CIs) were calculated. The variables showing significant associations with RFS or OS using the univariate analysis were included in the multivariate analysis. All statistical analyses were performed using the JMP Pro version 15 (SAS Institute, Cary, NC, USA). A *p* value < 0.05 was considered statistically significant.

## Results

### Patient characteristics

Clinicopathological characteristics of the patients with low or high NLR, PLR, CAR, and GPS values are summarized in Table 1. Compared with the other groups, there were more male patients in the high NLR group (*p* = 0.017). Liver tumor diameter was significantly larger in the high PLR, CAR, and GPS groups (*p* = 0.001, *p* < 0.0001, and *p* = 0.011, respectively). The percentage of patients receiving NAC was significantly higher in the high CAR group (*p* = 0.045).

**Table 1:** Clinicopathological characteristics of the patients according to the level of inflammatory indices

	Low NLR	High NLR		Low PLR	High PLR		Low CAR	High CAR		GPS: 0	GPS: 1/2	
	N=58 (%)	N=59 (%)	*P*-value	N=58 (%)	N=59 (%)	*P*-value	N=58 (%)	N=59 (%)	*P*-value	N=104 (%)	N=13 (%)	*P*-value
Sex			0.017*			0.401			0.220			0.292
Male	31 (53.4%)	44 (74.6%)		35 (60.3%)	40 (67.8%)		34 (58.6%)	41 (69.5%)		65 (62.5%)	10 (76.9%)	
Female	27 (46.6%)	15 (25.4%)		23 (39.7%)	19 (32.2%)		24 (41.4%)	18 (30.5%)		39 (37.5%)	3 (23.1%)	
Age (median, range), years	66 (38-81)	66 (33-83)	0.595	65.5 (33-81)	67 (41-83)	0.754	68 (33-83)	64 (38-83)	0.196	65.5 (33-83)	70 (45-83)	0.146
BMI (median, IQR)	21.9 (20.1-23.5)	22.1 (20.3-25.6)	0.460	23.2 (20.5-24.8)	21.2 (19.6-24.2)	0.096	22.5 (20-23.9)	21.8 (20.4-25.5)	0.639	21.8 (20.1-24)	23.6 (21-25.8)	0.204
Primary tumor location			0.955			0.439			0.720			0.355
Right sided	16 (27.6%)	16 (27.1%)		14 (24.1%)	18 (30.5%)		15 (25.9%)	17 (28.8%)		27 (26%)	5 (38.5%)	
Left sided	42 (72.4%)	43 (72.9%)		44 (75.9%)	41 (69.5%)		43 (74.1%)	42 (71.2%)		77 (74%)	8 (61.5%)	
NAC			0.521			0.162			0.045*			0.457
No	33 (56.9%)	37 (62.7%)		31 (53.4%)	39 (66.1%)		40 (69%)	30 (50.8%)		61 (58.7%)	9 (69.2%)	
Yes	25 (43.1%)	22 (37.3%)		27 (46.6%)	20 (33.9%)		18 (31%)	29 (49.2%)		43 (41.3%)	4 (30.8%)	
Preoperative CEA (median, IQR)	8.8 (3.7-19.7)	12.5 (3.9-45.2)	0.089	8.8 (3.7-19.8)	12.3 (5.1-47.9)	0.055	8.8 (.6-28.3)	12.2 (4.5-34)	0.263	9.4 (3.9-26.4)	1.3 (3.7-174.9)	0.192
Onset			0.778			0.646			0.646			0.896
Metachronous	27 (46.6%)	29 (49.2%)		29 (50%)	27 (45.8%)		29 (50%)	27 (45.8%)		50 (48.1%)	6 (46.2%)	
Synchronous	31 (53.4%)	30 (50.8%)		29 (50%)	32 (54.2%)		29 (50%)	32 (54.2%)		54 (51.9%)	7 (53.8%)	
Metastatic tumor location			0.074			0.306			0.619			1.000
Bilobar	27 (46.6%)	18 (30.5%)		25 (43.1%)	20 (33.9%)		21 (36.2%)	24 (40.7%)		40 (38.5%)	5 (38.5%)	
Hemilobar	31 (53.4%)	41 (69.5%)		33 (56.9%)	39 (66.1%)		37 (63.8%)	35 (59.3%))		64 (61.5%)	8 (61.5%)	
Liver tumor diameter (median, IQR)	25 (17.8-39.3)	25 (20-53)	0.197	24 (15-34.3)	33 (22-60)	0.0011*	23 (16.8-32.3)	38 (22-61)	<.0001*	25 (20-40)	60 (21.5-87.5)	0.011*
Tumor number			0.408			0.648			0.408			0.556
Solitary	29 (50%)	25 (42.4%)		28 (48.3%)	26 (44.1%)		29 (50%)	25 (42.4%)		47 (45.2%)	7 (53.8%)	
Multiple	29 (50%)	34 (57.6%)		30 (51.7%)	33 (55.9%)		29 (50%)	34 (57.6%)		57 (54.8%)	6 (46.2%)	

*BMI* body mass index, *CAR* C-reactive protein/albumin ratio, *CEA* carcinoembryonic antigen, *IQR* interquartile range, *GPS* Glasgow prognostic score, *NAC* neoadjuvant chemotherapy, *NLR* neutrophil/lymphocyte ratio, *PLR* platelet/lymphocyte ratio

### Comparison of RFS and OS between the low and high NLR, PLR, CAR, and GPS groups

**Fig. 2:**
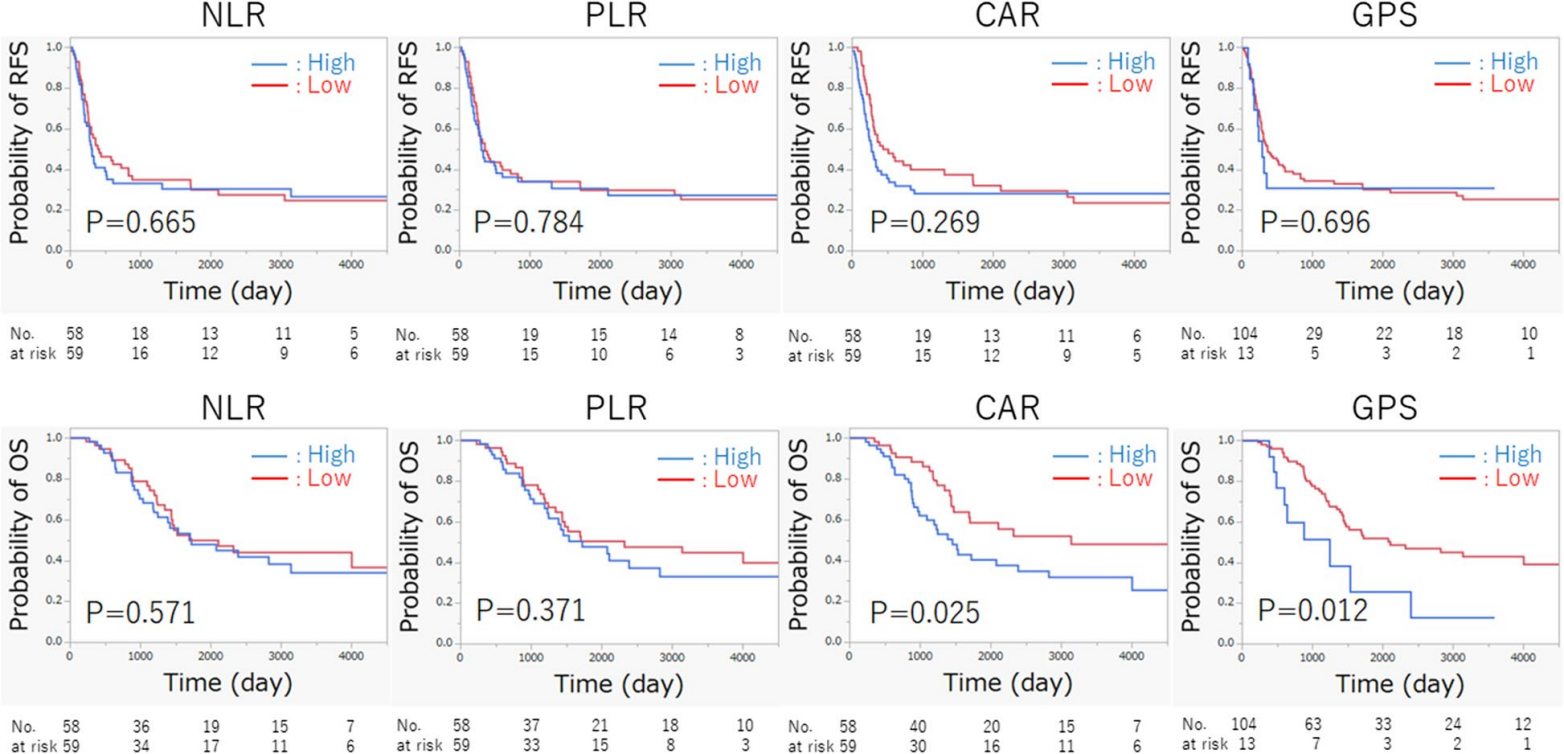
Kaplan–Meier curves of RFS and OS according to the levels of inflammatory indices. CAR, C-reactive protein/albumin ratio; GPS, Glasgow prognostic score; NLR, neutrophil/lymphocyte ratio; OS, overall survival; PLR, platelet/lymphocyte ratio; RFS, recurrence-free survival

The RFS and OS curves of the low and high NLR, PLR, CAR, and GPS groups are shown in Fig. 2. RFS curves were not significantly different between the groups. However, the low CAR and low GPS groups were associated with significantly better OS (*p* = 0.025 and *p* = 0.012, respectively). In the NLR and PLR groups, there were no significant differences between the two groups.

### Univariate and multivariate analyses for RFS and OS

The results of univariate and multivariate analyses for RFS and OS are shown in Tables 2 and 3.

**Table 2:** Univariate and multivariate analyses of recurrence-free survival

	N	Event	Univariate			Multivariate		
			95% CI	HR	*p*-value	95% CI	HR	*p*-value
**Sex**								
Female	42	26	1					
Male	75	52	0.74-1.90	1.12	0.473			
**Age**								
≥70	40	26	1					
<70	77	52	0.81-2.07	1.29	0.288			
**Primary tumor location**								
Left	85	55	1					
Right	32	23	0.71-1.89	1.16	0.558			
**NAC**								
Yes	47	37	1			1		
No	70	41	0.27-0.66	0.42	0.0002*	0.37-1.05	0.62	0.075
**Preoperative CEA**								
≥5	83	57	1					
<5	34	21	0.52-1.42	0.86	0.546			
**Onset**								
Synchronous	61	48	1			1		
Metachronous	56	30	0.23-0.59	0.37	<.0001*	0.28-0.78	0.47	0.0038*
**Tumor location **								
Hemilobar	72	43	1			1		
Bilobar	45	35	1.05-2.59	1.65	0.0292*	0.67-2.16	1.21	0.531
**Tumor diameter **								
≥50	23	17	1					
<50	94	61	0.42-1.23	0.72	0.223			
**Tumor number **								
Multiple	63	46	1			1		
Solitary	54	32	0.38-0.94	0.60	0.026*	0.53-1.80	0.98	0.952
**NLR **								
≥2.03	59	38	1					
<2.03	58	40	0.58-1.41	0.91	0.665			
**PLR**								
≥134	59	38	1					
<134	58	40	0.60-1.47	0.94	0.784			
**CAR**								
≥0.030	59	39	1					
<0.030	58	39	0.50-1.21	0.78	0.271			
**GPS**								
1/2	13	9	1					
0	104	69	0.43-1.75	0.87	0.696			

*CAR* C-reactive protein/albumin ratio, *CEA* carcinoembryonic antigen, *CI* confidence interval, *HR* hazard ratio, *GPS* Glasgow prognostic score, *NAC* neoadjuvant chemotherapy, *NLR* neutrophil/lymphocyte ratio, *PLR* platelet/lymphocyte ratio

**Table 3:** Univariate and multivariate analyses of overall survival

	**N**	**Event**	**Univariate**			**Multivariate**^a^					
			**95% CI**	**HR**	***p***-value	**95% CI**	**HR**	***p***-value	**95% CI**	**HR**	***p***-value
**Sex**											
Female	42	19	1								
Male	75	35	0.57-1.76	1.00	0.986						
**Age**											
≥70	40	17	1								
<70	77	37	0.60-1.91	1.07	0.809						
**Primary tumor location**											
Left	85	39	1								
Right	32	15	0.61-2.01	1.11	0.738						
**NAC**											
Yes	47	26	1			1			1		
No	70	28	0.34-0.99	0.58	0.045*	0.47-1.59	0.86	0.636	0.44-1.46	0.80	0.464
**Preoperative CEA**											
≥5	83	41	1								
<5	34	13	0.34-1.21	0.64	0.169						
**Onset**											
Synchronous	61	33	1			1			1		
Metachronous	56	21	0.29-0.87	0.50	0.014*	0.33-1.15	0.61	0.126	0.32-1.09	0.59	0.090
**Tumor location **											
Hemilobar	72	28	1			1			1		
Bilobar	45	26	1.09-3.20	1.86	0.024*	0.84-2.69	1.51	0.165	0.85-2.66	1.50	0.163
**Tumor diameter **											
≥50	23	12	1								
<50	94	42	0.33-1.21	0.63	0.165						
**Tumor number **											
Multiple	63	33	1								
Solitary	54	21	0.36-1.10	0.63	0.102						
**NLR **											
≥2.03	59	28	1								
<2.03	58	26	0.50-1.46	0.86	0.571						
**PLR**											
≥134	59	28	1								
<134	58	26	0.46-1.34	0.78	0.372						
**CAR**									―		
≥0.030	59	33	1			1					
<0.030	58	21	0.31-0.93	0.54	0.027*	0.33-0.99	0.57	0.048*			
**GPS**						―					
1/2	13	9	1						1		
0	104	45	0.20-0.84	0.41	0.015*				0.19-0.83	0.40	0.013*

^a^Adjusted for the following variables: NAC, Onset, Tumor location

*CAR* C-reactive protein/albumin ratio, *CEA* carcinoembryonic antigen, *CI* confidence interval, *HR* hazard ratio, *GPS* Glasgow prognostic score, *NAC* neoadjuvant chemotherapy, *NLR* neutrophil/lymphocyte ratio, *PLR* platelet/lymphocyte ratio

Univariate analysis showed that NAC (“no” versus “yes”; HR, 0.42; 95% CI, 0.27–0.66; *p* = 0.0002), onset (metachronous versus synchronous; HR, 0.37; 95% CI, 0.23–0.59; *p* < 0.0001), tumor location (bilober versus hemilober; HR, 1.65; 95% CI, 1.05–2.59; *p* = 0.029), and tumor number (solitary versus multiple; HR, 0.60; 95% CI, 0.38–0.94; *p* = 0.026) were prognostic factors for RFS. As per the multivariate analysis, onset (metachronous versus synchronous; HR, 0.47; 95% CI, 0.28–0.78; *p* = 0.0038) was an independent prognostic factor. The univariate analysis demonstrated that NAC (“no” versus “yes”; HR, 0.58; 95% CI, 0.34–0.99; *p* = 0.045), onset (metachronous versus synchronous; HR, 0.50; 95% CI, 0.29–0.87; *p* = 0.014), tumor location (bilober versus hemilober; HR, 1.86; 95% CI, 1.09–3.20; *p* = 0.024), the CAR (< 0.030 versus 0.030≤; HR, 0.54; 95% CI, 0.31–0.93; *p* = 0.027), and the GPS (0 versus 1/2; HR, 0.41; 95% CI, 0.20–0.84; *p* = 0.015) were the prognostic factors for OS. On multivariate analysis, CAR (< 0.030 versus 0.030≤; HR, 0.57; 95% CI, 0.33–0.99; *p* = 0.048) and GPS (0 versus 1/2; HR, 0.40; 95% CI, 0.19–0.83; *p* = 0.013) were the independent prognostic factors.

### Relationship between each inflammatory index and presence of TILs

**Fig. 3:**
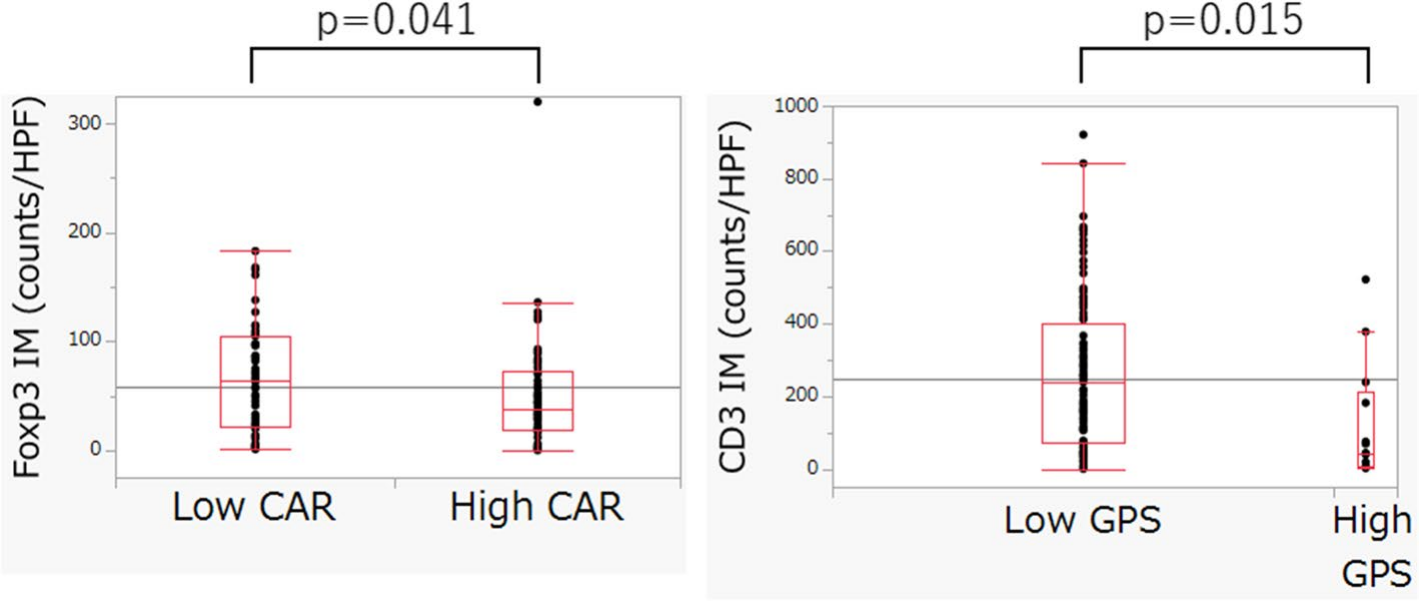
Relationship between the level of inflammatory indices and presence of tumor-infiltrating lymphocytes

We evaluated the relationship between each inflammatory index and the presence of TILs. Foxp3+, a marker of regulatory T-cells, was expressed more intensely in the IM in the patients with a low CAR (*p* = 0.041), and the number of CD3 + TILs in the IM was significantly higher in the low GPS group (*p* = 0.015) (Fig. 3). Differences in the number of other TILs between the groups were not significant (Additional files 1, 2 and 3).

## Discussion

In this study, we evaluated the utility of inflammatory indices, including the NLR, PLR, CAR, and GPS, in terms of the long-term outcomes of CRLM after hepatectomy. Clinicopathological features differed between the groups in terms of sex, tumor diameter, and percentage in patients who underwent NAC. The low CAR and GPS groups showed significantly better OS using the log-rank test. Furthermore, low values of the CAR and GPS were independent prognostic factors for better OS using univariate and multivariate analyses. Moreover, the low CAR group was related to high infiltration of Foxp3+ lymphocytes, and a low GPS was associated with a high number of CD3+ TILs.

Both the CAR and GPS depend on CRP and albumin levels. CRP is an acute phase protein secreted by hepatocytes in response to acute inflammatory stimuli through producing interleukin-1 (IL-1) and interleukin-6 (IL-6). Some cytokines, including IL-1 and IL-6, are released during cancer progression [19]. Thus, CRP levels may reflect tumor activation. Serum albumin level is an indicator of immune nutritional status. Hypoalbuminemia induces an impaired immune response, which in turn, promotes cancer growth. Additionally, decreased albumin levels demonstrate an increased inflammatory status with elevated levels of cytokines, such as tumor necrosis factor-alpha, IL-1, and IL-6, which may contribute to cancer progression [20]. Therefore, high CAR or GPS may reflect impaired tumor immunity and suggest tumor progression.

The present study showed that Foxp3+ and CD3+ lymphocytes were more commonly found in samples from the low CAR and low GPS groups, respectively. Nakayama et al. revealed an association between CRP and TILs in patients with renal cell carcinoma: higher CRP levels indicated a stronger infiltration of CD8+, Foxp3+, and CD163+ TILs [18]. Martin et al. showed that lower CAR levels were related to CD8+ TILs presence [17]. Foxp3+ lymphocytes have been reported to suppress anti-tumor immunity, which worsens survival [21, 22]. However, many studies have revealed that presence of Foxp3+ lymphocytes was associated with favorable prognosis in patients with primary CRC [12, 15, 16, 23]. Ladoire et al. described that a possible reason for this discrepancy may have been due to the characteristics of gut microbiota. They suggested that Foxp3+ lymphocytes may prevent bacteria-driven inflammation and carcinogenesis [24]. However, generally, there are no microbiomes in the liver. Alternatively, high Foxp3 expression has been associated with better OS in patients with hepatocellular carcinoma based on its capacity to inhibit the expression of the oncogenic protein c-Myc and induce apoptosis in tumor cells [25]. Thus, the association between high Foxp3 expression and good prognosis in patients with CRLM needs further research.

The International Study Group of Pancreatic Surgery recommended preoperative determination of the GPS in patients with borderline resectable pancreatic cancer [26]. The present study demonstrated that both CAR levels and the GPS had prognostic value for OS in patients with CRLM. Although the CAR is based on continuous variables such as CRP and serum albumin levels, the GPS depends on categorical variables. In the present study, only 11.1% of patients were categorized as having mGSP 1 or 2. Other reports on CRLM also showed that only 11–14% of cases were categorized as having GPS 1 or 2 [27, 28]. Thus, we suggest that CAR may sufficiently represent the systemic inflammatory status and have a good predictive value for survival in patients with CRLM.

In patients with CRLM, multidisciplinary therapy, including NAC, is pivotal. Therefore, effective predictors of chemotherapy or immune checkpoint inhibitors responses are warranted to prolong survival. Some researchers have reported that response to immune checkpoint inhibitors differed according to the number of TILs [29, 30]. However, TILs can only be evaluated using surgical specimens, and biopsy samples cannot overcome sampling errors or tumor heterogeneity. Our study indicated that some inflammatory indices were associated with the presence of TILs. This means that inflammatory indices, rather than TILs’ status, may be good predictors of response to neoadjuvant therapy.

The present study has several limitations. First, there might be a potential risk of selection bias due to the single-center retrospective design. Second, NAC was administered to some patients, which might have influenced systemic inflammatory status and presence of TILs. Third, other immune cells, such as CD4+, CD163+, PD-1+, and PD-L1+ TILs, were not evaluated in the present study, which could be helpful for a comprehensive understanding of the relationship between systemic inflammation and the tumor microenvironment. Finally, there might be problems about the weak statistical power in the present study. Using stricter *p* values such as with Bonferroni correction in the multiple hypothesis testing, as in our study, has been previously recommended. Additionally, in an analysis of statistical power, the effect size of the CAR was 0.19. Thus, the effect size was small and our results should be interpreted with caution.

In conclusion, the CAR and GPS are simple, inexpensive, and objective assessment tools for evaluating the inflammatory status in patients with CRLM and predicting survival. In addition, these tools can indicate presence of certain types of TILs in the IM of tumor.

## Supplementary Information

**Additional file 1.**

**Additional file 2.**

**Additional file 3.**

## Data Availability

All the data generated or analyzed during this study are included in this published article [and its supplementary information files].

## Abbreviations

CAR: C-reactive protein/albumin ratio
CEA: Carcinoembryonic antigen
CI: Confidence interval
CRC: Colorectal cancer
CRLM: Colorectal liver metastasis
CRP: C-reactive protein
HR: Hazard ratio
IM: Invasive margin
GPS: Glasgow prognostic score
NAC: Neoadjuvant chemotherapy
NLR: Neutrophil/lymphocyte ratio
OS: Overall survival
PLR: Platelet/lymphocyte ratio
TC: Tumor center
TILs: Tumor-infiltrating lymphocytes
RFS: Recurrence-free survival
