# Supplementary figures and images for: C-reactive protein/albumin ratio and Glasgow prognostic score are associated with prognosis and infiltration of Foxp3+ or CD3+ lymphocytes in colorectal liver metastasis
Source: BMC Cancer. 2022 Aug 1;22:839. doi: 10.1186/s12885-022-09842-4 (PMC9344720; doi:10.1186/s12885-022-09842-4)

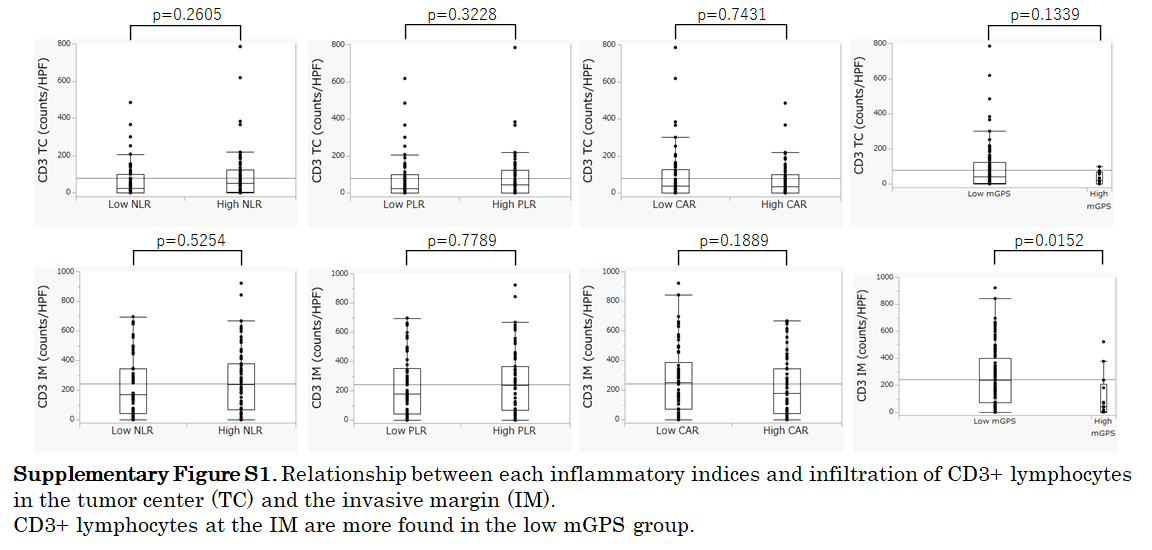

Supplement: Supplementary file 1 — Additional file 1. [file 12885_2022_9842_MOESM1_ESM.tif]

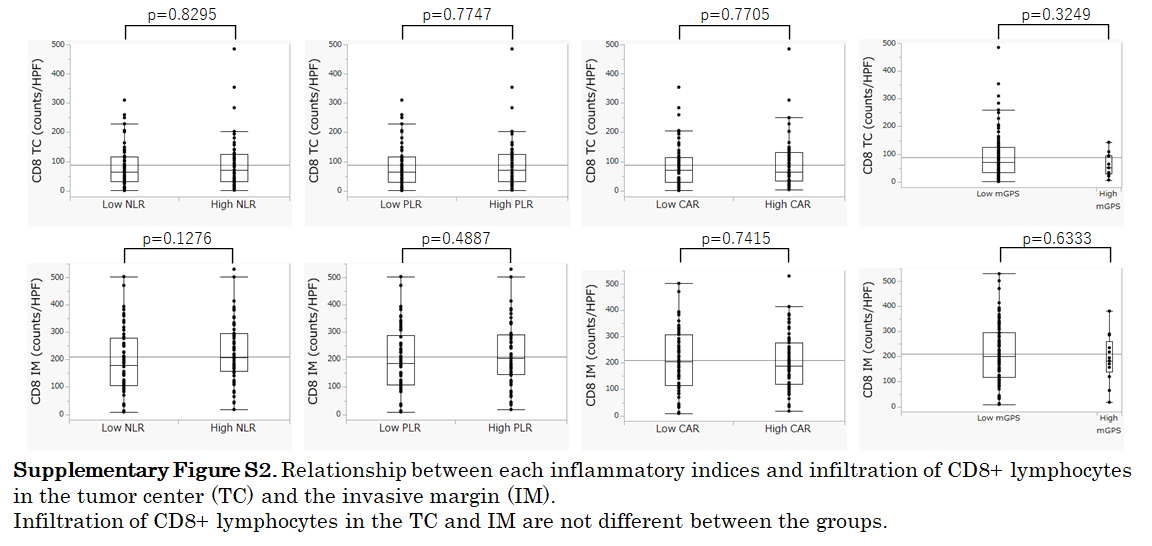

Supplement: Supplementary file 2 — Additional file 2. [file 12885_2022_9842_MOESM2_ESM.tif]

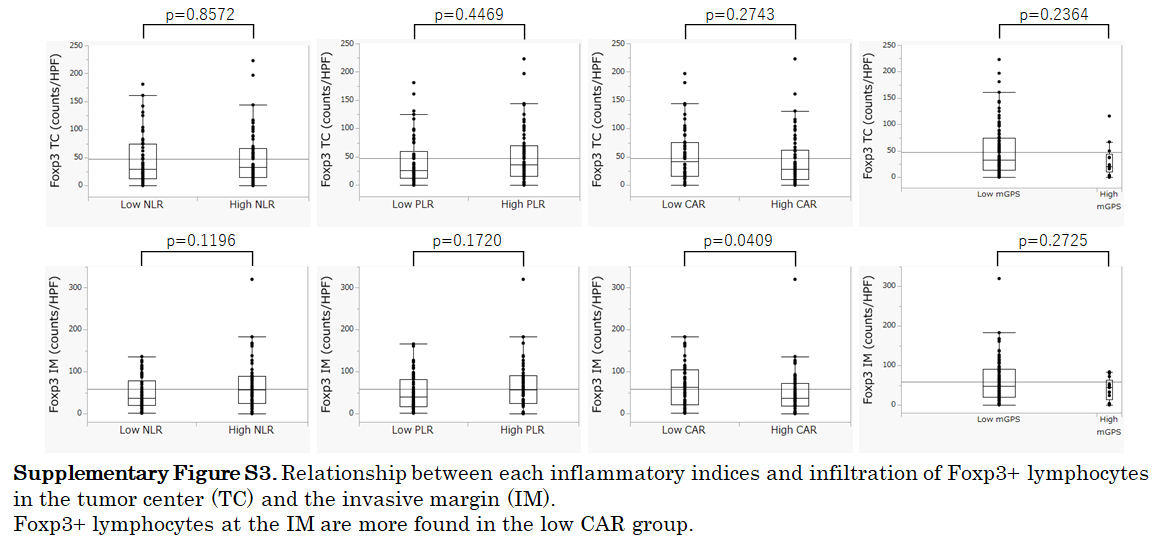

Supplement: Supplementary file 3 — Additional file 3. [file 12885_2022_9842_MOESM3_ESM.tif]
